# Evaluation of a symptom-based score in combination with CA125 to predict ovarian malignancy in women with adnexal mass

**DOI:** 10.1186/s43046-022-00111-w

**Published:** 2022-02-16

**Authors:** Amruthamshu Krishnamurthy, Jayalakshmi Durairaj, Murali Subbaiah

**Affiliations:** Department of Obstetrics and Gynaecology, JIPMER, Pondicherry, India

**Keywords:** Adnexal mass, CA125, Ovarian malignancy, Symptom-based score

## Abstract

**Background:**

Adnexal masses are a common problem seen in women. The aim of this study was to determine the appropriate cut-off for symptom-based score to predict ovarian malignancy in women with adnexal mass and to evaluate it in combination with CA125.

**Methods:**

This was a prospective study involving 341 women with adnexal mass who underwent surgery. A symptom-based scoring system was administered to the women, preoperatively, and CA125 levels were documented. Receiver operating characteristic curve (ROC) analysis was used to determine the appropriate cut-off for the symptom-based scoring. Results for this symptom-based scoring and CA125 were correlated with surgical pathological findings.

**Results:**

Out of the 341 women with adnexal mass, 112 were diagnosed to have ovarian malignancy. The mean age of women was 43.6±13.8 years. Using ROC analysis, symptom score ≥9 was determined to be the appropriate cut-off. The area under curve (AUC) at this cut-off score was found to be 0.87 (95% CI 0.83–0.91). The sensitivity, specificity, positive predictive value (PPV), and negative predictive value (NPV) at this cut-off was found to be 84.8%, 88.6%,78.5%, and 92.3%, respectively. Combining CA125 and symptom score resulted in higher sensitivity (96.4%) and NPV (97.4%) with specificity and PPV of 65.5% and 57.8%, respectively.

**Conclusion:**

Symptom score in combination with CA125 has good ability to predict ovarian malignancy in women with adnexal masses.

## Background

Adnexal masses are a common problem seen in women, with a lifetime risk of 5–10% [1]. Most of these adnexal masses are benign neoplasms, especially in premenopausal women. However, in postmenopausal women, 36–59% of these adnexal masses may be malignant [2]. The overall survival is less than 50% in women diagnosed to have ovarian cancer [3].

Several biomarkers and imaging tools have been evaluated in patients presenting with adnexal mass to rule out malignancy [4]. Cancer antigen 125 (CA125) is one of the most used and studied biomarker in such situations. But CA125 levels are elevated in only 50% of patients with stage 1 disease [5]. There is a need for better evaluation tools in patients presenting with adnexal mass.

Goff et al. devised a symptom index (SI) for ovarian cancer screening in primary care clinic [6]. It is a self-administered tool based on patients’ symptoms and their frequency and severity. Combination of biomarkers with SI has been found to increase their performance in predicting cancers in women presenting with adnexal mass [7, 8]. A new symptom-based scoring system has been recently described for use in primary care clinic [9]. This scoring system has not been evaluated in women with adnexal mass. This study was done to determine the appropriate cut-off for this score in women with adnexal mass and to evaluate it in combination with CA125 to predict ovarian malignancy.

## Methods

This was a prospective study conducted at a tertiary care hospital in India from January 2016 to October 2017. The study was approved by the Institute Ethics Committee for human studies. Informed consent was obtained from all the patients. Women ≥18 years presenting with adnexal mass and planned for surgery were included in the study. Exclusion criteria of the study were pregnancy and history of gynecologic cancer. Serum CA125 levels were measured in all patients before surgery. A symptom-based scoring system was administered to the patients preoperatively [9]. The scoring system includes 7 symptoms: abdominal bloating (score 2), urinary frequency (score 2), rectal bleeding (score 2), postmenopausal bleeding (score 2), loss of appetite (score 3), abdominal pain (score 3), and abdominal distension (score 5). The total score was calculated by adding all these scores. An additional 1 point was added to women aged ≥50 years. This scoring was done by one of the investigators who was blinded to the ultrasound findings and CA125 level. CA125 ≥ 35 U/mL was considered abnormal. The nature of adnexal mass was confirmed by histopathological examination report after surgery.

### Statistical analysis

Statistical testing was performed using STATA software Version 13.1(STATA Corp, Texas, USA). Continuous variables are presented as mean with standard deviation and categorical variables as percentages. Receiver operating characteristic curve (ROC) analysis was used to determine the appropriate cut-off for the symptom-based scoring in women with adnexal mass. The diagnostic ability of this symptom score in combination with CA125 was assessed by calculating their sensitivity, specificity, positive predictive value (PPV), and negative predictive value (NPV).

## Results

A total of 341 women with adnexal mass were included in the study after excluding 9 women (pregnancy in 2; prior history of ovarian malignancy in 7 women). The mean age of women was 43.6±13.8 years. Thirty-eight patients (11.1%) were nulliparous. The mean BMI was 22.1 ± 2.7 kg/m^2^. There were 141 (41.35%) post-menopausal women in the study cohort.

Final histopathological examination detected primary ovarian malignancy in 112 women (32.84%) as shown in Table 1. Three (0.87%) women had metastatic ovarian cancer, and 17 (4.98%) were found to have borderline ovarian tumor. ROC analysis was used to determine the appropriate cut-off for the symptom-based scoring to diagnose ovarian malignancy. The area under curve (AUC) at a cut-off score ≥9 was found to be highest; AUC 0.87 (95% CI 0.83–0.91). The sensitivity, specificity, PPV, and NPV at this cut-off was found to be 84.8%, 88.6%,78.5%, and 92.3%, respectively. Hence, this cut-off was chosen as the appropriate cut-off for women with adnexal mass. Score≥4 was found to have poor specificity (53.7%) and PPV (52%), although the sensitivity was 100%.

**Table 1:** Histopathological findings and cancer staging in 112 women detected to have primary ovarian malignancy

	Total (*n*= 112)	Premenopausal (*n*=49)	Postmenopausal (*n*= 63)
**Pathology,** ***n*** **(%)**			
Serous	66 (58.92%)	24 (48.97%)	42 (66.66%)
Mucinous	29 (25.89%)	11 (22.44%)	18 (28.57%)
Endometroid	6 (5.35%)	4 (8.1%)	2 (3.17%)
Clear cell	1 (0.89%)	1 (2.04%)	
Germ cell	7 (6.25%)	7 (14.28%)	
Leiomyosarcoma ovary	1 (0.89%)	1 (2.04%)	
SCT & GCT SP ovary	1 (0.89%)	1 (2.04%)	
Carcinoid tumor	1 (0.89%)		1 (1.58%)
**Stage,** ***n*** **(%)**			
I	29 (25.89%)	15 (30.61%)	14 (22.22%)
II	19 (16.96%)	6 (12.24%)	13 (20.63%)
III	52 (46.42%)	23 (46.93%)	29 (46.03%)
IV	12 (10.71%)	5 (10.20%)	7 (11.11%)

Symptom score ≥9 was found in 121 (35.48%) women and CA 125 ≥ 35 U/mL in 166 (48.68%) women. Table 2 shows the results of symptom score and CA125 levels according to pathologic diagnosis. Women who had symptom score ≥9 or CA125 ≥ 35 U/mL, or both were designated as high-risk women. The diagnostic accuracy of symptom score in combination with CA125 in detecting ovarian malignancy is shown in Table 3.

**Table 2:** Symptom score and CA125 levels according to pathologic diagnosis

		Benign (*n*= 209)	Borderline tumour (*n*= 17)	Ovarian cancer (*n*=112)	Metastatic (*n*= 3)
Symptom score≥9	Yes	15 (7.2%)	8 (47.1%)	95 (84.8%)	3 (100%)
	No	194 (92.8%)	9 (52.9%)	17 (15.2%)	0 (0%)
Ca125≥35U/mL	Yes	53 (25.4%)	14 (82.4%)	97 (86.6%)	3 (100%)
	No	156 (74.6%)	3 (17.6%)	15 (13.4%)	0 (0%)
Symptom score≥9 or Ca125≥35U/mL	Yes	60 (28.7%)	15 (88.2%)	108 (96.4%)	3 (100%)
	No	149 (71.3%)	2 (11.8%)	4 (3.6%)	0 (0%)

**Table 3:** Diagnostic accuracy of symptom score in combination with CA125 in detecting ovarian malignancy

	Sensitivity % (95% CI)	Specificity % (95% CI)	PPV %^a^ (95% CI)	NPV %^b^ (95% CI)
Symptom score≥9	84.8 (76.8–90.9)	88.6 (83.8–92.4)	78.5 (70.1–85.5)	92.3 (87.9–95.4)
Ca125≥35U/mL	86.6 (78.9–92.3)	69.9 (63.5–75.7)	58.4 (50.5–66)	91.4 (86.3–95.1)
Symptom score≥9 or Ca125≥35U/mL	96.4 (91.1–99)	65.5 (59–71.6)	57.8 (50.3–64.9)	97.4 (93.5–99.3)

^a^Positive predictive value

^b^Negative predictive value

## Discussion

In this prospective observational study involving 341 women with adnexal mass, 112 were diagnosed to have ovarian malignancy. Symptom score≥ 9 was found to be the appropriate cut-off score in them. Using this cut-off, the sensitivity, specificity, PPV, and NPV was found to be 84.8%, 88.6%,78.5%, and 92.3%, respectively. CA125≥ 35 U/ml was found to have slightly better sensitivity (86.6%) than the symptom score, but the specificity, PPV, and NPV were found to be lower. Combining CA125 and symptom score resulted in high sensitivity (96.4%) and NPV (97.4%) with specificity and PPV of 65.5% and 57.8%, respectively.

CA125, human epididymis protein 4(HE4), and OVA1 are commonly used biomarkers in women presenting with adnexal mass [10–12]. HE4 may have better sensitivity than CA125 in stage 1 ovarian cancer [13]. The sensitivity of OVA1 for epithelial ovarian cancers has been reported to be 99% [14]. However, these latest biomarkers may not be readily available in many developing countries. Symptom-based scoring system in combination with CA125 seems to be an attractive option in such a setting. Combining CA125 and symptom score resulted in high sensitivity (96.4%) in our study. This symptom-based scoring system in combination with CA125 can be used as a triaging tool to decide appropriate surgical care or referral for women with adnexal mass. If symptom score≥ 9 or if CA125≥ 35 U/ml, they can be referred to an oncology centre for better management of the disease. Several studies have shown that outcome in ovarian cancer is significantly improved when managed by gynecologic oncologists [15, 16].

The symptom score used in this study was developed by Grewal et al. for ovarian cancer screening in patients attending primary care clinic [9]. Symptoms of 212 women with ovarian cancer were compared with symptoms in 1060 age and practice-matched controls. The scoring system includes 7 symptoms and patients’ age. Using a score of ≥4 cut-off, the scoring system was found to have a specificity of 91.32% and sensitivity of 72.64%. In our study, we evaluated this scoring system in women with adnexal masses and found a score of ≥4 to have poor specificity. Score ≥9 was found to be a better cut-off. Using a cut-off ≥9, the sensitivity, specificity, PPV, and NPV were found to be 84.8%, 88.6%,78.5%, and 92.3%, respectively.

SI is another symptom-based scoring system developed for ovarian cancer screening in primary care clinic [6]. It consists of 6 symptoms (increased abdominal size, bloating, difficulty eating, or feeling full quickly and abdominal or pelvic pain). The SI is considered positive if these symptoms occur for more than 12 times a month and for a duration of less than 1 year. The main limitation of this scoring system is recall bias [17]. The scoring system described by Grewal et al. and used in this study eliminates this recall bias [9].

SI in combination with biomarkers has been evaluated in women presenting with adnexal mass to predict ovarian malignancy [7, 8]. A combination of SI and OVA1 was evaluated in 218 women who underwent surgery for pelvic mass in a prospective study [8]. The combination of SI and OVA1 was found to have a sensitivity of 100%, specificity of 36.3% with a NPV of 100%. They also evaluated a combination of SI and CA125 in these women. This combination was found to have a sensitivity of 96.9%, specificity of 59.7% with NPV of 97.3%. This is similar to the findings of our study where a combination of CA125 and symptom score was found to have a sensitivity of 96.4%, specificity of 65.5%, and NPV of 97.4%.

In another study involving 218 women, three markers (CA125, HE4, and SI) were evaluated in women with adnexal mass [7]. Patients were considered triple screen positive “if at least 2 of the 3 markers were abnormal” (CA125 ≥ 35 U/mL, HE4 ≥ 140 pmol/L, positive SI). The triple screen was found to have a sensitivity of 79%, specificity of 91%, and a NPV of 89%.

The strength of this study is that it is a prospective study and that the symptom score and CA125 levels were documented in all the patients. The relatively large sample size is another strength of the study. Investigators were blinded to the ultrasound findings and CA125 levels. This was done to eliminate observer bias. The limitation of the study is the high prevalence of ovarian cancer in our study population. As our centre is a tertiary referral hospital, this high prevalence was expected. The results of our study may not be applicable when dealing with a cohort with lower prevalence of ovarian malignancy. Further evaluation of this symptom-based scoring is needed in centres with lower prevalence of ovarian malignancy.

## Conclusions

Symptom score in combination with CA125 has good ability to predict ovarian malignancy in women with adnexal masses. Further studies are needed to confirm our findings in lower-risk population.

## Data Availability

The datasets generated and analyzed during the current study are not publicly available but are available from the corresponding author on request.

## Abbreviations

ROC: Receiver operating characteristic curve
AUC: Area under curve
PPV: Positive predictive value
NPV: Negative predictive value
CA125: Cancer antigen 125
SI: Symptom index
HE4: Human epididymis protein 4
